# Promoting Healthy Behaviors Among Adolescents and Young Adults With Intellectual Disability: Protocol for Developing a Digital Intervention With Co-Design Workshops

**DOI:** 10.2196/47877

**Published:** 2023-07-28

**Authors:** Ulrika Müssener, Pontus Henriksson, Catharina Gustavsson, Hanna Henriksson, Mårten J Tyrberg, Stefan Johansson, Kristin Alfredsson Ågren

**Affiliations:** 1 Department of Health, Medicine, and Caring Sciences Linköping University Linköping Sweden; 2 Center for Clinical Research Dalarna Uppsala University Falun Sweden; 3 School of Health and Welfare Dalarna University Falun Sweden; 4 Department of Public Health and Caring Sciences Uppsala University Uppsala Sweden; 5 Centre for Clinical Research Uppsala University Uppsala Sweden; 6 Region Västmanland Västmanland Hospital Västerås Sweden; 7 Division of Media Technology and Interaction Design School of Electrical Engineering and Computer Science Kungliga Tekniska Högskolan Stockholm Sweden; 8 Department of Design Sciences Lund University Lund Sweden

**Keywords:** intellectual disability, lifestyle behavior, eHealth, co-design, digital intervention, lifestyle, adolescents, young adult, healthy diet

## Abstract

**Background:**

Intellectual disability (ID) is a neurodevelopmental disorder associated with a poorer health profile and higher mortality. Young people with ID have more sedentary lifestyles than their typically developing peers. Consequently, this group is at significant risk of developing lifestyle diseases (ie, noncommunicable diseases) later in life. Increasing physical activity and eating a healthier diet have been argued to be effective ways to improve the health of adolescents and young adults with ID. Digital interventions are a viable option for improving health behaviors.

**Objective:**

This research protocol describes a co-design approach using workshops to develop a digital intervention that promotes healthy behaviors, including increasing physical activity and eating a healthier diet, among adolescents and young adults with ID.

**Methods:**

A participatory design using a co-design approach will be applied as a strategy to include potential users of the digital intervention and other stakeholders in the research process, comprising research design, data collection, and data analysis. A total of 7 to 10 workshops will be conducted aimed at developing a digital intervention and will include procedures for assessing needs; facilitators and barriers to health promotion; physical, mental, and social well-being; participation; and relationships. The workshops will include 12 to 18 stakeholders with experience of clinical practice and research related to young people with ID, including relatives, as well as adolescents and young adults (aged 16-25 years) with mild to moderate ID. Participants will perform a mixture of individual and group work using whiteboards, sticky notes, felt-tip pens, cards, balls, stickers, and wireframe templates. Data analysis will take place concurrently with data collection as an iterative process. Transcribed data from the audio and video recordings of the groups’ discussions will be analyzed following a qualitative methodological procedure.

**Results:**

This study protocol provides a systematic record of the scientific methodologies used when developing the digital intervention and provides insights into the potential practical solutions and challenges when following a co-design approach in which relatives and professionals, as well as adolescents and young adults with ID, are included as research partners. Recruitment of participants started in April 2023. Data collection, analysis, and reporting will be completed in December 2023.

**Conclusions:**

This study will explore the effectiveness of workshops at gathering rich, reliable, and valid data in a co-design approach with participants. The results will provide increased knowledge in how to use technology to develop novel, evidence-based, and scalable interventions that adolescents and young adults with ID can and want to use to motivate physical activity and a healthier diet. The project will provide a simple and cognitively accessible digital solution for promoting lifestyle behaviors tailored to the needs of adolescents and young adults with ID.

**International Registered Report Identifier (IRRID):**

PRR1-10.2196/47877

## Introduction

### Health of Adolescents With Intellectual Disabilities

Intellectual disability (ID) is a neurodevelopmental disorder with onset during childhood [1]. In Sweden, about 1% to 3% of the population is diagnosed with ID. ID differs from person to person but always impacts conceptual, social, and practical skills [2]. The ability of people with ID and the support they therefore need varies, as the severity of ID can be mild, moderate, or severe [1]. ID is associated with more health problems, poorer health profiles, and higher mortality rates [3]. People with ID are more likely to have a chronic illness, such as cardiovascular and respiratory disease [4] or mental illness [5,6]. Adolescence is a time of change, marking the transition from childhood to adulthood, and it is a critical life period when health-related behaviors often are set for adulthood [7]. Adolescents with ID have, in general, a more sedentary lifestyle, a less healthy diet, and greater social isolation compared to adolescents in the general population [3,8]. Consequently, this group is at significant risk of developing lifestyle diseases (ie, noncommunicable diseases), such as type II diabetes, hypertension, obesity, heart disease, stroke, and cancer later in life [4,9-11]. A recent population-based longitudinal cohort study of adolescents and young adults in Sweden found that premature mortality was significantly increased in those with ID [12].

### Digital Interventions to Promote Lifestyle Behaviors

As in the general population, lifestyle behaviors have a strong impact on health for this group [13]. Establishing healthy behaviors is often easier and more effective during adolescence than trying to change unhealthy behavior during adulthood [7]. Increasing physical activity and eating a healthy diet have been argued to be the most effective ways to improve the health of adolescents with ID [14]. However, few studies have investigated health promotion interventions that target this group [13]. One viable option for improving health behaviors is to use digital interventions, called eHealth, which is defined as the use of information and communication technologies for health and can include the use of email, text messages, push notifications, websites, and mobile apps [15]. Previous systematic reviews [16,17] and our own previous randomized controlled trials (RCTs) [18-20] have shown that eHealth provides a wide range of possibilities for health-behavior promotion among young people in the general population. Digital interventions may also be useful for adolescents and young adults with mild to moderate ID, but there is a lack of such studies. Research comparing the internet use of adolescents with ID with a reference group of adolescents without ID shows that 70% of adolescents with ID have a smartphone and use the internet for entertainment purposes, compared to over 95% among adolescents without ID [21]. This is in line with other research and provides the knowledge that digital devices and internet activities are used in this target group [22-24]. Although Johansson et al [25] reported that there is a disability digital divide related to cognitive understanding and language difficulties, there is a possibility to design usable and accessible digital interfaces by using simple and logical interaction, clear design, and easy-to-understand written and audiovisual language combined with the use of clarifying icons and symbols. Digital interfaces can also be constructed to be adaptable to personal needs [26] and interoperable with personal assistive technology [27]. Combined, these measures can create a cognitively accessible digital intervention [28,29]. However, few studies have examined the feasibility or effectiveness of eHealth interventions to promote healthy behaviors (eg, a healthy diet and physical activity) in adolescents with ID. Furthermore, few studies have examined the possibility of co-designing eHealth interventions together with, rather than for, adolescents and young adults with ID. Only one protocol study for an RCT [30] has used a digital intervention to encourage youth with ID to increase their physical activity, and that study also targeted adults with ID. Clearly, there is a need to develop and design interventions that promote healthy behaviors tailored to the needs of adolescents and young adults with ID.

### Participatory Design With a Co-Design Approach

The World Health Organization’s Global Strategy on Digital Health (2020-2025) [15] prioritizes the development and adoption of appropriate, acceptable, and scalable health solutions to promote health and well-being worldwide. Participatory design [31] with a co-design approach [32,33] is crucial for successful development of digital interventions, as it is an effective method to understand the context, culture, attitudes, behaviors, needs, and expectations of those for whom the digital intervention is being designed. Previous research, including our own studies targeting adolescents and lifestyle behaviors [18-20,34-36], has consistently shown that the active participation of stakeholders and end users throughout the design of digital interventions supports the development of an intervention that meets the needs of its intended users. The development of a complex intervention requires several phases, although these phases do not follow a linear sequence [37]. The developers and designers of eHealth interventions targeting people with ID should be aware that this group might have the same needs to stay or become healthy as anyone, but they will have special prerequisites to consider due to their intellectual challenges, and there will be special requirements on how to design and promote such an intervention [38,39]. Through the co-design process, all included persons are given the possibility to become active partners at different stages in the development process [31].

### Objective

The objective of this research protocol is to describe a co-design approach using workshops to develop a digital intervention that promotes healthy behaviors among adolescents and young adults with ID.

## Methods

### Study Context

This study is part of a larger research project that aims to develop and evaluate a digital intervention to promote healthy behaviors among adolescents and young adults with ID. To accomplish the purpose of the research project, we have established an ID research forum together with adolescents and young adults with ID; stakeholders, such as professionals within the social welfare, health care, and school systems; relatives; other relevant partners; and researchers. The development of the digital intervention will be based on a review of the literature and existing evidence and will be centered on insights of stakeholders and adolescents and young adults with ID gathered through specially adapted workshops [40].

### Study Design

A participatory design approach using co-design [41] will be applied, in which research is conducted together with participants instead of on them. Co-design will be applied as a strategy to include potential users of the digital intervention and other stakeholders in the research process, including research design, data collection, and data analysis. The work draws from value sensitive design [42], a theoretically grounded approach that includes ethical and moral values in the design of technology. Value sensitive design allows stakeholders to have a voice in the design process and uses a tripartite method to perform conceptual, empirical, and technological investigations. Adolescents and young adults with ID; stakeholders within the health care, social service, and school systems; designers; accessibility experts; and researchers will form a design team. The design team will use an iterative and nonlinear process to design solutions to complex and prespecified problems. Every co-design activity will be carefully planned to fit the need to involve adolescents and young adults with ID.

Workshops provide a platform that can aid researchers in identifying and exploring relevant factors in a given domain by providing a way to understand complex work and knowledge processes that are supported by technology; an example of this is eHealth [43]. Workshops have a 3-fold purpose and focus within research: as a means, as a practice, and as a research methodology [44]. This study focuses on the workshop as a means to do co-design and develop the process for participation and as a research method to generate and collect data.

### Setting, Recruitment, and Participants

Adolescents and young adults with ID, as well as stakeholders living in Östergötland, Sweden, will be invited to workshops. The inclusion criteria for taking part in workshops for adolescents and young adults with ID will be as follows: (1) diagnosis of mild to moderate ID; (2) age between 16 and 25 years; (3) frequent use of either smartphones, tablets, or computers, that is, digital literacy, since we want them to use that experience in designing a digital intervention; (4) willingness to participate in a workshop; and (5) ability to provide informed consent. An equal mix of genders will be sought. Workshops with stakeholders will be divided into 2 groups with the following participants: group 1, including professionals from the areas of social welfare, health care, and the school system, and group 2, including relatives and representatives from the disability organizations. The inclusion criteria for the stakeholders will be as follows: (1) experience with clinical practice or other experiences related to adolescents with ID; (2) willingness to participate in a workshop; and (3) ability to provide informed consent. All participants will be geographically located in the region of Östergötland. Participants will be recruited with purposeful sampling to provide a representative sample of all relevant areas in collaboration with research partners in Östergötland and already established networks of the authors.

### Data Collection

**Figure 1:**
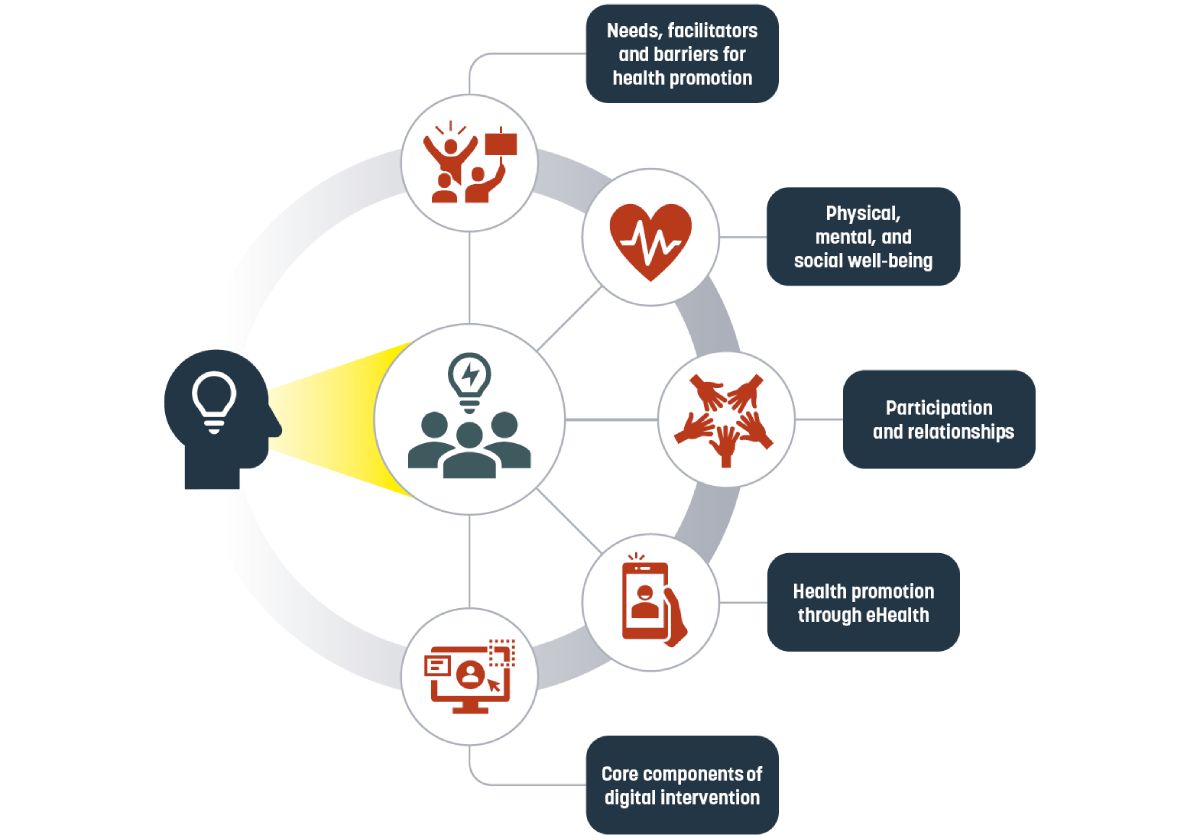
An overview of the included themes in the co-design workshops.

In total, 7 to 10 workshops [43] will be conducted between May and August 2023. Young adults and adolescents with ID will participate in a series of 3 workshops. The co-design process with specially adapted workshops will be used to gather rich, reliable, and valid data involving all participants. An overview of the included themes in the workshops is presented in Figure 1. The following themes will be included in all workshops: needs; facilitators and barriers to health promotion; physical, mental, and social well-being; participation and relationships; health promotion through eHealth; and core components of digital intervention. All workshops will be conducted in a meeting room at Linköping University or at rooms close to where young people with ID go to school or after-school clubs. The workshops will be organized in rooms with a whiteboard, internet connection, and wall surfaces suitable to tape up sheets of paper or sticky notes. Each workshop will produce rich data material, including design artifacts such as sketches, wireframe templates, screenshots of participants’ phones, and lists of technological barriers and enablers. The workshops will also be documented with video and audio recordings and field notes, allowing participants to revisit important discussions and ideas. All collected data will be used in an iterative analytical process. The data will be used to evaluate the workshop as a tool for co-designing together with people with ID and to design the digital intervention for promoting a healthy lifestyle. Each workshop will last approximately 2 hours, including breaks.

### Structure and Activities of the Workshops

A structure of the workshops has been outlined. The workshop phases, including preparation, introduction, the main act, and evaluation, as well as planned activities, are presented in Table 1 and further described below. Workshops with relatives and representatives from disability organizations and professionals will be initially conducted once per group of stakeholders. Adolescents and young adults with ID will take part in 3 consecutive workshops.

**Table 1:** Structure and activities of the workshops.

Phase	Structure and activities
Preparation	Provide information to participants (information videos, information letters, informed consent forms) Prepare workshop management (moderator, notetaker, video documenter) Construct the workshops (structure, content, and length) Prepare workshop materials (sticky notes, felt-tip pens, balls, wireframe templates of a mobile phone, stickers, colored cards) Prepare the use of assistive technology (devices for time management, taking turns, and recording) Prepare surveys
Introduction	Joint meal Presentations given by participants Moderators introduce the workshop and the timetable Notetaker and video documenter present assistive devices
Main act	Moderator introduces the topics to discuss and to work with Individual and group work (using whiteboards, sticky notes, felt-tip pens, cards, balls, stickers) Identifying core components using own devices and wireframe templates Tasks using colored cards to indicate agreement on suggestions (green, yellow, red) Survey on demographic variables and expectations on participating in co-design workshops (only for participants with intellectual disability)
Evaluation	Qualitative evaluation of the participants’ perceptions of their experiences taking part in the workshop Quantitative evaluation survey: demographic variables and perceptions of participation in the co-design process (only for participants with intellectual disability)

#### Preparation

The workshops will be carefully planned in line with the aim of the study. In preparation for participation in the workshops for adolescents and young adults with ID, a short video will be produced by the researchers in cooperation with relatives and representatives from the disability organizations. The video will be shown to the participants to explain the aim of the research project and ethical issues. All participants will be informed that the purpose of the workshops is to address barriers and facilitators to health promotion and to co-design components of a digital intervention. To avoid dropping out caused by, for example, issues with time management or memory issues, we will reach out and contact participants to remind them of the upcoming workshop. Workshop management will be organized to divide roles (eg, moderator, notetaker, and video documenter) between researchers. Workshop materials and assistive technology will be prepared, including cognitive adaptation to involve adolescents and young adults with ID.

#### Introduction

The workshops with participants with ID will be executed according to set procedures to make them cognitively accessible: start the workshop by establishing a convivial tone, present the agenda and the upcoming tasks, and present the rules for collaboration. Furthermore, to respect the timetable for the workshop, we will find a good pace and rhythm for the work, allow room and space for everyone to participate (while respecting participants who temporarily want to be silent), and give all participants time to think and reflect; we will stop and explain things that participants do not understand. We will start all workshops with a meal together to equalize differences in social status, get to know each other, and make participants feel comfortable. The participants will also give presentations. All participants (adolescents and young adults with ID; professionals from the areas of social welfare, health care, and the school system; and relatives and representatives from the disability organizations) will give oral presentations, preferably incorporating their own drawings. Moderators will introduce the workshops, including their length, schedule, and breaks. The notetaker and video documenter will explain the assistive devices.

#### Main Act

The moderators will present the proposed topics to discuss and work with during the workshop. The topics are (1) the health and well-being of young people with ID, (2) support for health-related activities and behaviors, and (3) the core components to include in a digital intervention to promote health behaviors. The co-design process will be implemented by giving all included participants the possibility to become active partners in all parts of the main act phase of the workshop. The goal is to both discuss the topics and create new outputs (ideas, concepts, and designs), as well as to evaluate special aspects of interest, such as drawing upon ideas for the design of a digital intervention prototype to promote healthy behaviors. Throughout the workshop, participants will make use of graphical arrangements along with verbal exchanges to express their ideas and to discuss proposals. Participants will perform a mix of individual and group work using whiteboards, sticky notes, felt-tip pens, cards, balls, and stickers. The selection of the specific materials and tools will be made in order to facilitate all participants to express themselves and hence to ensure a rich selection of data. The method of using a mix of individual work and group work encourages less talkative participants to have as much influence as more talkative participants. Additionally, the design team (ie, participating individuals and researchers) will use their own devices to discuss the apps they use that day. We will also use wireframe templates of a mobile phone for identifying core components of a digital intervention to promote health behaviors. Participants with ID will use stickers to prioritize what they perceive to be the most important components to include in a digital intervention and use green, yellow, and red cards to indicate if they agree with upcoming suggestions. An initial survey on demographic variables and expectations on participating in a co-design workshop will be provided to participants with ID.

#### Evaluation

The workshop will end with an evaluation of the participants’ perceptions of their experiences of taking part in the workshop. In line with the co-design process, all included participants will be active oral partners in a qualitative evaluation process. Further, during the last of the 3 workshops, participants with ID will also be provided with a quantitative survey on demographic variables, their perceptions of the co-design process as implemented in the workshops, and their interest in continued participation in our research.

### Data Analysis

Inspired by Sanders and Stappers [32], we use the following definition of co-design: “Co-design in a broader sense [refers] to the creativity of designers and people not trained in design working together in the design development process.” In co-design, roles are diversified and potential end users are given the position of “experts of his/her experiences” and play a central role in knowledge development, idea generation, and concept development. In order for them to take on this role, appropriate tools for ideation and expression are necessary [32], which in this study will be provided throughout the co-design workshops. Co-creation will be applied across the whole span of a design process. Data analysis will be performed together and take place concurrently with data collection. By striving for concurrent data collection and data analysis, we will start the joint analysis simultaneously during the workshops. The co-design process will be an iterative process between ideation and visualizing the ideas as frameworks for a preliminary prototype of the intervention. Data collection and data analysis are, in other words, seen as an intertwined back-and-forth process used by the design team. This process draws from methods adapted from the participatory action research and participatory design approach presented by Johansson [45] and will follow the double diamond design process with discovery, define, develop, and delivery phases [46]. The discovery phase will focus on understanding relevant phenomena and discussing the prerequisites for health promotion through a digital intervention. The define and develop phases will narrow the ideas down to those that will be developed in this project. The delivery phase will end with the framework for a preliminary prototype that the design team will use to move forward, but we will also work in iterative loops, going back and forth to co-design the prototype. This will be done by, for example, checking with participants, reflecting on feedback, and examining the processes of the included activities together with the participants. Preanalysis of quantitative aspects, for example, summarizing the colored-dot stickers, will be performed together by counting the dots and analyzing the results.

In order to analyze the workshop as a method for co-designing together with people with ID, transcribed data from the audio and video recordings of the groups’ discussions will be analyzed in an approach inspired by inductive program theory development [47] following a qualitative methodological procedure [48]. As suggested in literature describing participatory design methods from process to outcome [49], we will strive to follow an approach that creates space for all partners to draw on their different, yet complementary, experiences and skills and to determine their respective analytical roles and responsibilities. Analyses of the transcribed data will therefore be led by the first author (UM) and performed together with the researchers and representatives from the design team. Given the time and skills required, the transcribed data will be analyzed initially by the researchers. The evaluation survey will be analyzed using descriptive statistics. The representatives of the participating stakeholders will be actively engaged in determining the meaning of the results and their implications for action. By capturing visual details from the filmed workshops, this analytical approach will allow us to review not only what we said, but how we expressed it and in what situations. Systematic analyses will follow a prescribed sequential process [50].

### Ethical Considerations

All participants will receive written and oral information about the study, including information that participation is voluntary and that they are allowed to ask questions and leave the study at any time without giving reasons as to why. Informed consent will be obtained from all participants before participation in either written form or orally during audio recording. The study data will be deidentified before publication. Young adults and adolescents with ID will receive a diploma and a gift card of 100 SEK (US $9.77) for their participation. All procedures were approved by the Swedish Ethical Review Agency (Dnr 2022-06682-01).

## Results

Recruitment of participants started in April 2023 through initial contacts with schools, after-school clubs, community-based services, and social services. The recruitment continued until the end of June 2023. Initial workshops were planned for May 2023. Data collection and analysis are planned to be completed in September 2023. The main contribution of this protocol article is a detailed description of how to conduct and analyze co-design workshops to develop a digital intervention that promotes healthy behaviors among adolescents and young adults with ID. Full results from the workshops are expected to be published in December 2023.

## Discussion

This research protocol describes a co-design approach using workshops to develop a digital intervention that promotes healthy behaviors, such as physical activity and a healthy diet, among adolescents and young adults with ID. In this study protocol, we set out to describe a co-design approach using workshops to develop a digital intervention that promotes health-related activities and behaviors among adolescents and young adults with ID, who are a group that is often marginalized in society and in research regarding aspects of their own lives. However, research with the involvement of people with ID has proven to be plausible [51]. Through the co-design process, all included persons will be given the possibility to become active partners at all different stages in the development process. A description of how the participants will be involved in the co-design process has been presented. In this study protocol, needed adaptations are described and will be continuously redesigned to improve the involvement of people with ID in the co-design process.

It is well known that young people with ID have a more sedentary lifestyle compared to their typically developing peers. Specifically, they are less physically active, have a poorer diet, and are more socially isolated [3,8]. These health conditions are more prevalent due to lack of knowledge of their effects, reduced physical and behavioral skills, and lack of motivation attributed to ID [52]. As little evidence-based knowledge is available about how to promote physical activity, a healthy diet, and social participation for this group, this proposed study will identify needs, as well as facilitators and barriers to health promotion activities enabled through digital interventions. Furthermore, since few studies have examined the effectiveness of eHealth interventions to promote healthy behavior (eg, a healthy diet and physical activity) among adolescents and young adults with ID, our research aims to develop an eHealth intervention that targets this often-marginalized group. Including young individuals with ID in trials can involve challenges in many aspects of the research process. Common challenges in trials involving these individuals include informing the individuals with ID about the research process, obtaining informed consent, arranging transportation to research sites, and ensuring that instructions are followed [53]. Hence, it is particularly important to plan and conduct intervention studies for this group carefully. Therefore, by using a co-design approach when developing the intervention, we believe that we also will optimize participation in the trial and the potential uptake and reach of the digital tool.

This research project will develop an eHealth intervention that is specifically designed to address the needs of adolescents and young adults with ID. The digital intervention will be complex, especially given the variety of the intervention components needed, the degree of flexibility required, and the ability to adapt to the contextual, social, and practical skills of the individual. This research protocol describes a participatory design in order to develop a digital intervention to promote healthy behaviors. First, this protocol provides a systematic record of the scientific methodologies used when developing the intervention to enhance transparency of research and ensure research rigor. Second, the protocol provides insights into potential practical challenges and solutions when following a co-design approach in which relatives, professionals, and adolescents and young adults with ID are included as research partners. Third, this protocol provides procedural considerations on how to use workshops as a means and research method when developing digital interventions. The protocol describes how data will be collected and analyzed in cooperation with stakeholders and adolescents with ID. We hope this study will contribute to and inspire new approaches to develop effective interventions for lifestyle behaviors for young people with ID. Further studies will examine the effectiveness of our individually tailored digital intervention to support healthy lifestyle behaviors among adolescents and young adults with ID. If successful, the project will provide a simple and accessible digital solution for promoting physical activity and a healthy diet. Hence, our research has potentially important implications for both this target group and their support networks. Since it is necessary to engage stakeholders for widespread clinical implementation, this study will also be valuable during the later stages of our project. We have established networks through our ID research forum that include adolescents and young adults with ID, stakeholders (ie, professionals within the social welfare, health care, and school systems), relatives, other relevant partners, and researchers.

Several procedures will be performed to fulfill the quality criteria (ie, credibility, transferability, confirmability, dependability) for qualitative research to verify trustworthiness [54] when designing, analyzing, and reporting the processes related to the video-recorded workshops. The research process will be clearly described and documented. The Consolidated Criteria for Reporting Qualitative Research (COREQ) 32-item checklist [55] will be followed to create a clear audit trail. Data will be analyzed in accordance with the steps following indicative program theory development [47] and qualitative methodological procedures [48] to facilitate a rigorous and systematic process. The sample size will be based on data saturation [56], and data collection will accordingly be finalized when the data reach satisfactory depth and complexity to answer the aim with sufficient confidence. Trustworthiness will be further enhanced by the use of a guide [48] during all workshops. Video recordings will yield more details and secure high accuracy since the recorded data can be reviewed indefinitely [57]. Moreover, video recordings can help guard against researcher bias because they can be analyzed by researchers other than those who led the workshops [43]; hence, researcher triangulation will be used [48]. A possible limitation of the study design is that the transferability of the forthcoming results to other study contexts may vary depending on how the care is organized. However, we consider that the results from the study will be relevant and applicable to the development of digital interventions in similar settings. We expect that the results from this study will be valuable for the development of eHealth interventions in young adults with ID.

## Abbreviations

COREQ: Consolidated Criteria for Reporting Qualitative Research
ID: intellectual disability
RCT: randomized controlled trial

## Data Availability

The data sets generated during and/or analyzed during this study are not publicly available due to Swedish ethical review guidelines but are available from the corresponding author on reasonable request.
